# Advice for Improving the Experience of Web-Based Patient Portals: Qualitative Interviews With Caregiver-Adolescent Dyads

**DOI:** 10.2196/72134

**Published:** 2025-09-11

**Authors:** Rachel E Granberg, Jessica M Goldberg, Alison L Antes, Christine Bereitschaft, Fabienne Bourgeois, James DuBois, Bryan A Sisk

**Affiliations:** 1 Division of Hematology/Oncology, Department of Pediatrics Washington University School of Medicine St Louis, MO United States; 2 Washington University School of Medicine St Louis, MO United States; 3 Bioethics Research Center, General Medical Sciences, Department of Medicine Washington University School of Medicine St Louis, MO United States; 4 Division of General Pediatrics Boston Children's Hospital Boston United States; 5 Department of Pediatrics Harvard School of Medicine Boston, MO United States

**Keywords:** web-based patient portal, electronic health records, adolescent health care perspectives, qualitative research, adolescent, caregivers

## Abstract

**Background:**

Web-based patient portals can benefit adolescents and their caregivers by increasing access and providing greater understanding of one’s health information, enhancing communication with clinicians, and supporting caregiver influence. Despite these benefits, adolescent uptake has been low with high attrition rates. Feedback from adolescents and caregivers is essential to improve the uptake and usability of the web-based patient portal.

**Objective:**

The aim of the study is to identify advice for medical informatics administrators and clinicians directed at improving the adolescent and caregiver experience with the web-based patient portal.

**Methods:**

Caregivers completed a demographic survey followed by separate qualitative, semistructured interviews with adolescent-caregiver dyads with and without chronic illnesses. Caregivers and adolescents were interviewed separately regarding advice for administrators and clinicians on several topics, including (1) providing adolescent and caregiver portal access, (2) how doctors should discuss the portal with families and the content of their notes, and (3) what general advice they had to improve portal access for families. We performed thematic analysis to develop a codebook, and team members applied these codes to the transcripts and analyzed for overlaps and contrasts.

**Results:**

We performed 102 interviews with 51 dyads of caregivers and adolescents (26 with chronic illness and 25 without chronic illness). The majority of adolescents and their caregivers were White (adolescents: n=28, 55% and caregivers: n=28, 55%) or Black (adolescents: n=21, 41% and caregivers: n=21, 41%) and female (adolescents: n=30, 59% and caregivers: n=50, 98%). The majority of caregivers had accessed their child’s portal (n=33, 65%), whereas the majority of adolescents had not (n=17, 33%). We identified three themes related to adolescent and caregiver advice: (1) encouraging and supporting portal use, (2) recognizing the emotional experience of portal use, and (3) improving portal usability, understandability, and individualization. Adolescents and their caregivers provided specific recommendations regarding initial access or enrollment including improving resources and clinician encouragement as well as improving the usability in terms of user-friendly design, understandable language, and clear expectations. Finally, caregivers and adolescents had varied opinions on confidentiality and access but emphasized the importance of understanding the emotional impact and providing guidance to caregivers and adolescents.

**Conclusions:**

Adolescents and caregivers outlined critical advice to medical informatics administrators and clinicians to improve the patient portal uptake and usability. Further research is required to determine the best application for these recommendations, including the potential use for advance technologies to simplify results and clinical documentation, strategies to improve the user-friendly design, and how clinicians should communicate with families now that clinical documentation is viewable to patients and caregivers.

## Introduction

Web-based patient portals are resources that allow patients to access their electronic health information (EHI) and communicate with their clinicians through portal messages. Availability of portals has been bolstered by the 21st Century Cures Act, which was signed into US law on December 13, 2016. This law prohibits US health care systems and electronic health record (EHR) vendors from information blocking, “a practice [by a health care clinician or health IT developer, exchange, or network] that except as required by law or covered by an exception ... is likely to interfere with access, exchange, or use of EHI” [1]. This portion that expanded transparency in health care and access to EHI went into effect on April 5, 2021. Additionally, portal use surged during the COVID-19 pandemic when electronic communication, including video visits, increased, spurring uptake on a national scale [2].

In pediatrics, caregivers can access their child’s EHI through a proxy version of the portal. When children reach the age of adolescence (ranging from 10 to 16 years, depending on the US state) [3], most health care systems also provide adolescents with the option to register for their own portal account. However, each US state has laws that dictate which adolescent health information must be protected, usually related to sexual and reproductive health, mental health, and substance use disorders [4]. Each pediatric health care system must determine how to comply with these laws, and caregiver access may be limited to varying degrees when a child becomes an adolescent. As such, the usefulness of the caregiver proxy portal is often in tension, with restrictions required to ensure confidentiality of the adolescent’s sensitive, protected information.

Providing portal access can benefit both adolescents and their caregivers by improving understanding of one’s health, empowering adolescent self-management and autonomy, enhancing communication with clinicians, and supporting caregiver influence [5]. These benefits of improved medical competency and autonomy are especially critical for adolescents who are beginning to transition to adulthood and self-management. However, many adolescents demonstrate limited interest in using their portal [5], and their use has been low with studies citing variable rates of uptake between 15% and 64% [6-10]. In those who do use the portal, there is a high attrition rate, with one study finding that over 50% of portal accounts were actively used for less than 1 year [8].

Health care systems and administrators must consider devising strategies to simultaneously increase the usefulness of adolescent and caregiver proxy portals, support adolescent interest in using the portal, and maintain confidentiality of sensitive information. Input from adolescents and caregivers will be essential to inform these strategies, but few studies have explored these perspectives in depth. Most of this literature has focused on protecting confidentiality and increasing enrollment [11-16]. In this analysis, we aimed to address this gap by identifying adolescent and caregiver advice for medical informatics administrators and clinicians directed at improving the web-based patient portal experience.

## Methods

We report this study following the COREQ (Consolidated Criteria for Reporting Qualitative Research) guidelines [17].

### Participants and Recruitment

We conducted semistructured interviews with caregiver and adolescent dyads who received care in St Louis. We stratified sampling for adolescents with and without chronic illnesses and purposively sampled for racial and ethnic minorities and fathers. Adolescents with chronic illness were recruited from specialty clinics at a major academic medical center, including sickle cell disease (SCD), inflammatory bowel disease (IBD), and diabetes mellitus (DM). We chose these 3 chronic illnesses because they create lifelong medical complications and represent diverse racial and ethnic populations. Adolescents without chronic illness were recruited from 3 pediatric clinics and 1 adolescent medicine clinic associated with the academic medical center.

### Patient Portal Policies

All clinics used the same patient portal (called MyChart) with the same requirements for creating portal accounts. This portal allows participants to view scheduled appointments, clinician notes, and diagnostic results, communicate with their providers or clinics, and request medication refills, among other functions. Children aged 11 years and younger cannot have their own MyChart account, and caregivers may obtain full access to a proxy account by completing a proxy access form. When a child turns 12 years, caregivers lose proxy access by default. Adolescents can create their own account if they call the help desk during business hours. To provide proxy access for parents of adolescents, caregivers need to obtain signed forms of approval by the adolescent and the patient’s primary doctor. Every child in a family requires a separate proxy portal account with a unique password. There was no ability to link accounts of family members or to link accounts across institutions that used a different patient portal. There is no phone app available, though individuals can log in through an internet browser on their phone or computer.

### Data Collection

As part of a larger qualitative study [5], we performed semistructured interviews, in which we asked adolescents and caregivers to provide advice for administrators and clinicians regarding (1) providing adolescent and caregiver portal access, (2) how doctors should discuss the portal with families and write their notes, and (3) what general advice they had to improve portal access for families (Multimedia Appendix 1). This guide was informed by literature review, our prior work, and a stakeholder advisory board. BAS, CB, and another team member performed interviews between May and December 2022 via telephone or Zoom videoconferencing software (Zoom Video Communications) based on participant preference. Caregivers and adolescents were interviewed separately and privately. Interviews were audio-recorded and professionally transcribed. We collected demographics via a Qualtrics survey that was sent via email to caregiver participants.

### Data Analysis

BAS, CB, and JMG developed a codebook following validated phases of thematic analysis [18]. Coding involved multiple iterative steps: (1) read transcripts to familiarize themselves, (2) descriptively coded 5 transcripts to formulate preliminary codes, (3) grouped codes into categories and collapsed categories into representative themes, and (4) refined definitions for themes through 3 cycles of independent coding and consensus meetings. After reviewing 20 adolescent transcripts and 19 caregiver transcripts, we reached saturation for representative themes.

CB and REG independently applied this codebook to all transcripts using Dedoose qualitative software, reviewed each other’s application of codes, marked disagreements, and resolved disagreements via discussion. Subsequently, REG reviewed transcripts for each dyad, identifying overlaps and contrasts between caregivers and adolescents, as well as overlaps and contrasts for adolescents with a chronic medical condition and their caregivers compared to adolescent-caregiver dyads who do not have a chronic medical condition. Quantitative data on patient-reported demographics were analyzed using SPSS Statistics (version 29.0.2.0; IBM Corp).

### Ethical Considerations

The institutional review board at Washington University approved this study (202203147). We obtained informed consent from caregivers and assent from adolescents. All transcripts were deidentified prior to analysis. Participants were provided with a US $40 electronic gift card for their participation in the interviews.

### Positionality Statement

BAS is a pediatric oncologist and communication researcher with training in qualitative research. REG is a fellow in pediatric hematology/oncology with a master’s degree in public health and prior training in qualitative research. CB is a research coordinator with a master’s degree in social work and prior qualitative experience. Another team member who performed interviews has a master’s degree in public health and qualitative methods experience. JMG was a medical student with training in qualitative research. All team members have prior experience in qualitative research.

## Results

### Demographics

There were 102 separate interviews performed with 51 dyads, averaging 25 (SD 11) minutes. The majority of adolescents and their caregivers were White (adolescents: n=28, 55% and caregivers: n=28, 55%) or Black or African American (adolescents: n=21, 41% and caregivers: n=21, 41%), non-Hispanic (adolescents: n=46, 90% and caregivers: n=47, 92%), and female (adolescents: n=30, 59% and caregivers: n=50, 98%). Many caregivers had accessed their child’s portal (n=33, 65%), but fewer adolescents had accessed their portal (n=17, 33%; Table 1).

**Table 1:** Parent and adolescent dyad demographics.

Characteristics				No chronic illness (n=26)		Chronic illness (n=25)
**Parent**						
	Age (years), mean (SD)			44 (4.8)		46 (6.1)
	**Sex, n (%)**					
		Female	26 (100)		24 (96)	
		Male	0 (0)		1 (4)	
	**Race, n (%)**					
		Black or African American	9 (35)		12 (48)	
		Native Hawaiian or Pacific Islander	1 (4)		0 (0)	
		White	15 (57)		13 (52)	
		Other	1 (4)		0 (0)	
	**Ethnicity, n (%)**					
		Hispanic	4 (15)		0 (0)	
		Non-Hispanic	22 (85)		25 (100)	
**Adolescent**						
	Age (years), mean (SD)			15 (1.33)		15 (1.26)
	**Sex, n (%)**					
		Female	16 (62)		14 (56)	
		Male	8 (31)		10 (40)	
		Nonbinary or third gender	2 (7)		1 (4)	
	**Race, n (%)**					
		Black or African American	9 (35)		12 (48)	
		White	15 (57)		13 (52)	
		Other	2 (7)		0 (0)	
	**Ethnicity, n (%)**					
		Hispanic	4 (15)		1 (4)	
		Non-Hispanic	22 (85)		24 (96)	

### Encouraging and Supporting Portal Use

#### Improving Accessibility

Caregivers reported significant challenges with the enrollment processes for the proxy portal. These caregivers advised that the entire procedure should be easier, stating that the experience was “confusing” and requesting that more information be provided. Both caregivers and adolescents reported that the login process could be easier, stating the requirements for adolescents to have their own email made accessing the portal difficult. In addition, the sign-up process for the adolescent proxy portal required the adolescent to call the help desk during business hours, which conflicted with school and sports schedules. Further, institutions had rules about required characteristics of passwords, such as numbers and symbols, that frustrated some participants. Adolescents and caregivers thought it would be beneficial to have increased resources to assist in the sign-up and usability of the portal platform, including a website, pamphlet, or an individual that could guide families through the process.

#### Clinician’s Role in Educating and Promoting Use

Caregivers and adolescents acknowledged that the portal should be “brought up more” (Adolescent 26, no chronic medical condition) by the clinician to encourage use, with one caregiver stating that it should be “a part of the visit every time” (Caregiver 26, no chronic medical condition). Only one respondent stated that they did not know “if that’s really a doctor’s responsibility to worry about the portal” (Caregiver 13, IBD). Some felt it would be useful for clinicians to “be more informational” (Caregiver 46, SCD) about the portal components and sign-up process, with one person stating that they felt the doctor should have experience using the portal to better understand the patient perspective. Others recommended that clinicians outline acceptable uses of the platform, including types of portal messages that are acceptable and how to best communicate with them.

#### Recognize the Importance of the Portal for Chronic Illness

Nearly all caregivers and adolescents acknowledged the importance of respecting and protecting adolescent confidentiality. However, many caregivers of children with chronic illness described the importance of retaining access to nonsensitive information in the portal, such as notes, appointments, laboratory results, and medication lists. These caregivers worried that limiting parental access to this actionable information could be “very detrimental” (Caregiver 51, SCD), especially because adolescents are often not mature enough to navigate health care on their own (Table 2).

**Table 2:** Illustrative participant quotes on encouraging and supporting portal use.

Theme and subtheme		Illustrative quote
**Improving accessibility**		
	Easy sign-up	“The sign-up process took a while. It was kind of confusing. I think I had to have a code or something and I didn’t get it, so I remember that was kind of difficult. Making it just easier to actually get an account” [Caregiver 4, DM^a^].
	Easy access	“Just make it very simple to access it, not having to go through a lot of steps and give parents really good instructions on how to access it” [Caregiver 10, DM].
	Improved resources	“Maybe if it’s your first time using it, you do a step-by-step page or whatnot to show you where everything is and the layout” [Adolescent 47, no chronic medical condition]. “I think there needs to be better instruction and explanation on how to set it up, why it has to be set up that way. A lot of people just want you to, oh, set up the portal, but no one explains that you need a special code. You need consent. You need—so it’s never explained very well” [Caregiver 45, no chronic medical condition]. “If there’s a place that could have helped us, like a phone number we could’ve called, someone that could have walked us through it besides the doctor’s office themselves, that would’ve been more helpful too” [Caregiver 26, no chronic medical condition].
**Clinician’s role in educating and promoting use**		
	Educate	“I think that my doctors have been very good about telling us about the medical records, but again, I think they should just give a more precise knowledge about how to access the records” [Adolescent 41, no chronic medical condition]. “I think that they should just tell the family about it. Maybe they could show them on their phone. It’s like a demo, so it doesn’t show any personal stuff. Like they could show them on their phone, so they could see what the app looks like and show how user-friendly it is” [Adolescent 25, SCD^b^]. “I’m not sure that doctors understand how to use it. I think they need to have used it themselves, logged in, checked it out, seen it from a patient view, and—I don’t know—have better detailed description and notes of how to get in, who to call, what to do” [Caregiver 45, no chronic medical condition].
	Promote	“I would suggest the doctor tell the family that they should regularly look at the online medical records. Just so they know everything, all the information that’s on there that they need to know. If there’s something serious or even if there’s not, just to make sure that everything’s okay” [Adolescent 40, no chronic medical condition]. “Oh, I think they should definitely encourage it with every patient because it is a useful tool, and I thought should be presented. It’s not just information. It’s a useful tool that you can use to help take an active part in the health of the child” [Caregiver 5, IBD^c^].
	Outline acceptable uses	“Educating parents and teenagers, this is how you access it, this is what it’s good for, this is a positive use of it, and this is what you shouldn’t use it for” [Adolescent 20, no chronic medical condition]. “Just making clear how they prefer to communicate and how they want us as the patients to communicate with them or how they will choose to communicate. ‘I will send you a message through MyChart.’ We just recently went to our endocrine appointment and the nurse practitioner said, ‘I will call you.’ He did, he called and left a message because that was all clear. He told us how he was gonna communicate. Now that we do have MyChart, we have the phone, we have all these different ways and the after-hours communication just to be clear how they want, what method they would prefer, and what’s most effective for them” [Caregiver 2, DM].
**Recognize the importance of the portal for chronic illness**		
	—^d^	“I think it’s a good idea to let parents access it because they need to know what’s going on with their child’s health, and if they don’t, then their child could worsen their own health without them knowing” [Adolescent 16, SCD]. “I would tell them that these are minor children, and it is very important that parents are an integral role in the health of their children, and that they should have access to all of it” [Caregiver 12, IBD].

^a^DM: diabetes mellitus.

^b^SCD: sickle cell disease.

^c^IBD: inflammatory bowel disease.

^d^Not applicable.

### Recognizing the Emotional Experience of Using the Portal

#### Mitigate Stress Through Discussion and Resources

To decrease stress, caregivers advised that clinicians discuss with families the maturity level of the adolescent prior to granting access to ensure that the adolescent is ready for access. Caregivers also recommended that adolescents should be walked through the portal at their first time of access. A few adolescents worried about portal access being problematic for parents who were “helicopter parents” or overbearing. These adolescents advised that clinicians discuss the types of information that might be released through the portal, prepare them for potentially stressful results, and encourage them not to become fixated on every abnormal result. One caregiver and one adolescent noted the importance of maintaining the option for verbal communication with clinicians outside of the portal, especially when worrisome test results are released.

#### Modify Note Content

Adolescents and caregivers advised that notes should be tailored to remove and protect confidential discussions that occurred between the adolescent and their clinician. Some adolescents and caregivers advised a collaborative discussion between clinicians and adolescents about what specifically should be excluded from the notes to ensure confidentiality tailored to the comfort level of that patient and the patient-caregiver dynamic. Another caregiver suggested the clinician inform the adolescent that the confidential portion would not be included in the note to build trust.

Caregivers and adolescents also had recommendations for the content and language that were included in the portal. Many caregivers and adolescents stated the importance of considering adolescents’ emotions when writing notes, which involved using professional language and “stay[ing] positive and to stay just on facts. Maybe not put personal thoughts” (Caregiver 4, DM). A few participants discussed concerns that comments about the adolescent’s weight could have a negative emotional effect. These participants advised clinicians to be “sensitive and mindful” (Caregiver 42, no chronic medical condition) and use “very gentle phrasing” (Adolescent 23, no chronic medical condition). One adolescent reflected that they had “a doctor make jokes about me in my medical notes before ... It just made me not trust the doctor really, and withhold information from them because I didn’t wanna be judged about it” (Adolescent 10, DM) (Table 3).

**Table 3:** Illustrative participant quotes on recognizing the emotional experience of using the portal.

Theme and subtheme		Illustrative quote
**Mitigate stress through discussion and resources**		
	Prepare parents	“Then to the parents, be like, ‘If you see something that’s worrisome, don’t become overbearing,’ because I definitely think, especially with mental health, a step in the right direction is telling the doctor—maybe not your parent, but telling the doctor or someone else that you trust, and I definitely think if a parent becomes super overbearing or super controlling because they have access to these records, there’s the chance that the child won’t give that information and won’t be very open with their doctor. Then that could spill out into a bigger problem down the road” [Adolescent 29, no chronic medical condition]. “I think there’s maybe a possibility with other teenagers of parents who are striving for those perfect numbers, and that can put a lot of stress on children, so I think maybe in terms of the parents’ access, and it may not be restricting access to anything, but it could just be reminders that parents have lots of impact on their children, obviously, even if a child doesn’t have a condition, but especially with children that have a medical condition. Parents have a very big impact on how their child will view their health, and so I think that could be a good reminder to parents as well” [Adolescent 2, DM^a^].
	Communication of big news	“A worry I would have is if they have this big disease or something, just something big, something that’s a very big deal, it might be best to first tell them and then give the medical records” [Adolescent 23, no chronic medical condition]. “I feel like if there’s something that is detrimental, knowing that either an adolescent or a parent has access to that, that should be a verbal conversation versus seeing something like that written. You know what I mean? When you have that information, when you see that information, there are so many questions that go through your mind, and I think that would be unfair to the patient to have to read that and not be able to access the doctor immediately to have a discussion about that information” [Caregiver 27, no chronic medical condition].
**Modify note content**		
	Protect confidentiality	“I don’t think they should be putting things that they ask the parents to leave the room for because if they had the parents leave the room, they’re trying to get the teenager to be completely truthful and completely comfortable. Maybe I don’t think it’s the best idea for them to put that information in the Doctor’s Note, unless maybe it’s like a serious issue, and they got permission from the teenager during the doctor’s appointment” [Adolescent 3, DM].
	Sensitive language	“Very gentle phrasing, not saying this child is overweight. They need to work out now, something like that. Not that way, but something more like kind, considering people’s feelings and stuff” [Adolescent 23, no chronic medical condition]. “I would hate to see in a doctor’s note somethin’ that could be hurtful by the patient, where they need to lose a certain amount of weight by this time or they’re gonna have horrible—they’re gonna develop diabetes” [Caregiver 1, DM].

^a^DM: diabetes mellitus.

### Improving Portal Usability, Understandability, and Individualization

#### Improving Interpretation of Laboratory Results and Notes

For interpreting laboratory results, adolescents and their caregivers noted that having a dictionary, glossary of medical terms, or additional information such as reference values would be beneficial. Some specifically stated that without these resources, they resort to web-based searches to interpret results. Adolescents without chronic medical conditions did not have advice regarding improving laboratory understanding for individuals accessing the portal, presumably because they had few or no laboratory values in their medical records. Regarding clinician notes, many adolescents and caregivers described a “language barrier” (Adolescent 2, DM) due to the medical terminology and complexity of notes. They advised that notes should be understandable to the average person, with “everyday language instead of medical terms” (Adolescent 13, IBD). One caregiver specifically advised how clinicians’ notes could be a “bridge from the labs or the records” (Caregiver 8, IBD), with notes providing an explanation for difficult-to-understand laboratory results. Others advised that the notes did not need to change but that clinicians should speak with adolescents and their caregivers about the readability of these notes. A few adolescents and caregivers had suggestions regarding the content of notes, stating that they should contain next steps, address the concerns of the caregiver and patient, and be transparent and detailed.

#### Improving Portal Design

Some adolescents and caregivers reflected that the patient portal should be overall “user-friendly” (Caregiver 30, SCD) and “making sure that everything is findable, that needs to be found” (Caregiver 28, no chronic medical condition) with an organized layout. Caregivers of children with a chronic medical condition noted the potential benefits of having the office and doctor’s contact information “in case we need to get ahold of them quickly” (Caregiver 9, IBD). Several adolescents noted that “it would be more acceptable if it was an app” (Adolescent 46, no chronic medical condition).

#### Individualizing the Interface

Caregivers and adolescents recommended improving the level of customization of portal contents for adolescents and their caregivers. Suggestions included having a simpler interface for adolescents, having a more summarized note personalized to the adolescent, or personalizing the interface with users selecting for desired or undesired features.

#### Linking Accounts

Caregivers and adolescents recommended a feature by which portal accounts could be linked, such that caregivers could have one sign-in for all children or family members. Participants noted that this would ease usability and access, which can be hindered by requiring multiple logins and passwords. Caregivers also recommended an additional feature where records could be linked and shared across institutions, which would enable the streamlining of medical care (Table 4).

**Table 4:** Illustrative participant quotes on improving portal usability, understandability, and individualization.

Theme and subtheme			Illustrative quote
**Improving interpretation of laboratory results and notes**			
	Dictionary	“In bloodwork, there are a lot of medical terms that teens might not know. Try to make it easier for people to access information on that ... Well, maybe by the term, like white blood cell count for example, you just have a little info button that you can press it and it pops up some information about what that is” [Adolescent 5, IBD^a^].	
	Understandable language in notes	“Just make it as simple and as easy to understand and follow as possible. Maybe if they’re using medical terminology, to explain what that medical terminology means. What condition is going on, where it is affecting them physically, and treatment. Very specific and easy to understand” [Caregiver 10, DM^b^].	
	Note as a bridge to understand laboratory results	“I think they should be thorough and in layman’s terms. I think the labs can be, obviously, more medical terms, but the doctor’s notes should be the bridge from the labs or the records to better—to make it easier to understand” [Caregiver 8, IBD].	
	Prepare families for medical terminology	“I think they should write them according to how they were trained to write notes. I don’t think they should alter how they were taught to write notes. Write ’em as they should, but also let families know that these—prep ’em they contains medical terminology” [Caregiver 32, no chronic medical condition].	
**Improving portal design**			
	User-friendly	“Probably go to a graphic designer, somebody that designs websites, and just make an easily accessible app. Also make it look clean, I guess, because that makes you want to use it more” [Adolescent 46, no chronic medical condition].	
	Organized layout	“Maybe if there’s a way to organize it to where even if a teenager, even if I was there without my mom, I would be able to understand where to find things and just make it easier to understand where everything is. I think that would help” [Adolescent 13, IBD].	
	Contact information	“I think adding the doctors’ contact information for their office right there with their name in case we need to get ahold of them quickly” [Caregiver 9, IBD].	
	App form	“If they can make an app that would be easier to use” [Adolescent 7, DM].	
**Individualizing the interface**			
	Individualized notes	“Maybe they can make like a note for the kid, the teen, and a note for the parent. The note for the parent could be more in detail and stuff, and the note for the kid could be more summarized so they understand” [Adolescent 25, SCD^c^].	
	Simpler interface	“When I open the website, it makes sense to me, but it would be overwhelming to a child. Looking at the menu and the visits and the messages, and test results, scheduling appointment, those are things that—maybe it would just be like a little more child-like. To say a smiley face, you’re seeing your pediatrician in two weeks, how are you feeling? To be able to—instead of saying, ‘Send a message’ ‘Is there anything you wanna ask your doctor?’” [Caregiver 15, no chronic medical condition].	
	Personalized interface	“I would consider is having multiple sign-ons for each patient. That way, my son could have his sign-on. I could have my sign-on. Then if, let’s say, we both make it clear that he only wants to see certain things, his level could be set for that, but then I could see everything or vice versa. That way, it’s specific to what each user needs to see” [Caregiver 5, IBD].	
**Linking accounts**			
	Caregivers can link family member accounts	“They should make it so that the parents could have one account. Then the kids can come out of that account, where the parent doesn’t have to log in with the other kids. ’Cause in my family, I have four other siblings, so my mom is gonna have to deal with that and having four other accounts” [Adolescent 25, SCD].	
	Link across institutions	“That’s confusing that every institution has their own MyChart. If there was a way to make ‘em universal that they flow in together, that would be more helpful to the families and the patients” [Caregiver 53, no chronic medical condition].	

^a^IBD: inflammatory bowel disease.

^b^DM: diabetes mellitus.

^c^SCD: sickle cell disease.

## Discussion

### Principal Findings

Using the technology acceptance model framework, we consider the 2 key factors of perceived ease of use and perceived usefulness that influence the attitude, behavioral intention, and actual portal system use [19]. Adolescents and caregivers provided advice on how to improve the perceived ease of use, including improving the accessibility of portal enrollment and access, the understandability of its contents, and mitigation methods for potential emotional distress that could result from portal use. Regarding its perceived usefulness, while some recognized the usefulness and importance of the portal, many stressed that the perceived usefulness could be bolstered by clinician promotion of the portal and by improving the understandability and customization of its contents. There were three overarching themes that permeated the adolescent and caregiver recommendations: (1) encouraging and supporting portal use, (2) recognizing the emotional experience of portal use, and (3) improving portal usability, understandability, and individualization.

Increasing resources for accessing, using, and understanding the portal was nearly universally recommended. Areas that were identified to benefit from increased resources were establishing portal access and initial use as well as improving the understandability of laboratory results and clinician notes. This is particularly salient when discussing portal design for the adolescent age range, as the target audience’s maturity level and reading skills evolve over time. Some resources could be standardized, such as handouts to understand the enrollment process, a tutorial to demonstrate display features, or a glossary that explains basic medical terms. Other aspects, such as interpreting radiology reports or understanding the potential impact of an abnormal laboratory result, may require a more nuanced and personalized approach. Improvement on these more nuanced aspects could involve efforts to improve communication with clinicians or the incorporation of advanced technologies, such as large language model (LLM)–powered chatbots. Prior work demonstrated that LLMs can improve the readability of radiology reports [20,21]. Another study found that LLMs were able to generate accurate and comprehensive responses with minimal risk of harm to radiation oncology patient care questions, though with a higher-than-recommended readability level [22]. Additionally, LLMs can provide inaccurate information or generate “hallucinations,” which “include a vast array of failures of faithfulness and factuality” of the LLM [23]. More research, particularly in its application to the pediatric and adolescent population, is required to determine the safety, acceptability, and usefulness of LLMs in supporting portal use.

This study further emphasizes and expands on the idea that clinicians should be discussing patient portals with families. While clinician endorsement can influence portal engagement [24,25], this study is novel in identifying other important roles of clinician communication for the portal experience, including setting up parameters for confidentiality, alleviating emotional distress, and outlining expectations and communication preferences. Many caregivers and adolescents had recommendations for how clinicians may improve communication by way of their notes. These recommendations included using sensitive language that recognizes the potential emotional impact, using layman terms, and removing confidential information [26]. This research underscores the importance of clinician communication in promoting appropriate use and enhancing the effectiveness of the patient portal. Furthermore, it raises difficult questions about the purpose and audience of clinical notes. Should these notes primarily be written for medical documentation and communication with other clinicians, for understandable communication with caregivers and patients, or for liability or billing purposes? The same note might not be able to fulfill these divergent purposes [27].

Many caregivers and adolescents provided recommendations on improving the layout, as well as how EHR vendors could improve portal engagement and use by focusing on a user-centered design, which seeks to adapt the design and functionality of the portal based on user feedback and goals. Research in adult populations has found that identifying user goals and interests and then tailoring portal design and function to meet these needs improves engagement in the portal [28,29]. Furthermore, as research in adolescent and adult populations shows that portal engagement is associated with better medical adherence and understanding, as well as a better perception of control over one’s health, this engagement could have significant downstream effects [30-33]. Given that many adolescents show limited interest in using the portal [5-8], engaging in user-centered design might increase the perceived usefulness and ease of use of portals, which are central drivers of uptake and acceptance of novel technologies [34].

### Strengths and Limitations

This study had several limitations. First, while we purposively sampled for racial and ethnic minorities, as well as fathers, our study was limited by excluding non-English speakers and having few Hispanic participants and few fathers as participants. Therefore, these perspectives may not be appropriately reflective or generalizable to these populations. Second, adolescents with greater concerns about privacy and confidentiality might have been less inclined to participate in this study, which may result in a decreased emphasis on this aspect compared to a more generalized adolescent population. Third, as patients were recruited from a single health care system in St Louis, MO, using a single EHR platform, the results may not be generalizable to other regions of the United States or to other hospital systems using a different EHR platform.

This study also had several strengths. First, the unique perspective of this qualitative research evaluates not just the barriers and benefits of portal engagement for adolescents and their caregivers, but uniquely identifies potential solutions. Second, while the study was limited in having few fathers, few Hispanic participants, and excluding non-English speakers, it still represents a diverse population by including both adolescents and caregivers, those with and without chronic medical illnesses, and purposively sampling for racial and ethnic minorities. This improves generalizability by providing advice for clinicians and administrators that serves a broader group of patients and families, particularly when considering those with versus those without a chronic medical condition, who may have different priorities when using the portal.

### Conclusions

Caregivers and adolescents had specific recommendations for administrators and clinicians to improve the usefulness of the patient portal. Adolescents and caregivers universally recommended more resources for accessing and using the portal, improved clinician communication regarding the portal, and enhanced user-centered design of the portal. Clinicians can take steps to better understand and inform families about portal access and functions. However, the onus lies with administrators and EHR vendors to make structural changes to the portal user interface and functionalities that can improve the usefulness and usability of the portal. The advice provided in this study should guide these improvement efforts.

## Appendix

Multimedia Appendix 1 Interview guide.

## Abbreviations

COREQ: Consolidated Criteria for Reporting Qualitative Research
DM: diabetes mellitus
EHI: electronic health information
EHR: electronic health record
IBD: inflammatory bowel disease
LLM: large language model
SCD: sickle cell disease

## Data Availability

The datasets generated or analyzed during this study are available from the corresponding author on reasonable request.
