# Supplementary material for: Advice for Improving the Experience of Web-Based Patient Portals: Qualitative Interviews With Caregiver-Adolescent Dyads
Source: JMIR Pediatr Parent. 2025 Sep 11;8:e72134. doi: 10.2196/72134 (PMC12464496; doi:10.2196/72134)
Supplement: Multimedia Appendix 1 [file pediatrics_v8i1e72134_app1.docx]

**Interview Guide Draft – Adolescent Interviews**

**[Background on 21^st^ Century Cures Act]**

First, I’d like to tell you a little about what we’ll talk about today. Across the country, teenagers can now access their medical records and doctors notes through online portals. At our hospital, we call this portal *MyChart.* These medical records include things like doctors notes, lab results, and imaging results. Parents are given different types of access at different hospitals.

Even though hospitals are offering this access to teenagers, there is no agreed upon best practices. So different hospitals have different rules for how they give access to teenagers and their parents. Today I am going to ask a lot of open-ended questions about your thoughts on this medical record access for teenagers and parents.. There are no wrong answers, and you are the expert on your thoughts and beliefs. I just want to learn as much as I can from you.

**[Adolescent’s Health]**

1. To get us started, could you tell me a bit about your health? Do you have any medical problems?

***[If so]***

- 1. What types of medical care do you need?
  2. Which types of doctors do you see regularly?
  3. How often do you have medical appointments?
  4. What types of care do you need at home?

**[EHI Access for Adolescent]**

1. The online medical record contains lab values, imaging results, appointment reminders, and doctors notes, among other things. Have you ever logged into your *MyChart* portal and looked at your medical record?

***[If yes – Reviewed EHI]***

- 1. How did you use the portal? What did you look at?
  2. What made you want you to look at your medical record?
     1. What were you hoping to learn?
     2. How did this help you?
     3. What problems did this cause for you?
  3. What made it harder to review the medical record online?
     1. What could have made accessing your online medical record easier?
     2. What could have made the online medical record more helpful for you?
  4. Did you ever read any of the notes from your doctor’s visits?

**[If yes – Yes Notes]**

- - 1. What types of notes did you read?
       1. What made you want to read these notes?
    2. What were you hoping to learn from the notes?
       1. How did this help you?
       2. Whatwere the downsides of reading these notes?
       3. What could have made these notes more helpful?
    3. Did reading these notes make you look at your team differently?
    4. How did reading these notes affect the way that you saw yourself in relation to your care?

**[If no – No Notes]**

1. Were you aware that you could read these notes?
2. What kept you from reading the doctors notes?

***[If No – Did Not Review EHI]***

- 1. Were you aware that you could access your online medical record?
     1. What kept you from accessing your online medical record?
     2. Now that you know you can use the portal, do you think you will in the future?
  2. Were there times when it would have been helpful to access your medical record through the portal?
     1. How would this have been helpful?

1. Overall, do you think that teenage patients should be able to access their online medical records? [If so – At what age]
   1. Do your views change based on the child’s age [**assuming <18 years**]?
   2. What are the potential benefits of patients like you accessing their medical records?
   3. What are the potential downsides of this access?
2. Should teenagers be able to read their doctors notes?
   1. What are the potential benefits of teenagers reading their doctors notes?
   2. What are the potential harms of providing teenagers with this access?
   3. In what circumstances would you definitely want to read your doctors notes?
   4. In what circumstances would you definitely **not** want to read your doctors notes?
3. **[If healthy adolescent**] How do you think your views would change if you had a chronic, serious illness like diabetes or sickle cell or inflammatory bowel disease?
4. **[If chronically ill adolescent]** How do you think your views would change if you did not have a [list disease]?

**[Access for Parents]**

1. Now I want to switch gears and think about parents. Do you think your parents have accessed your medical records through the portal?
   1. How did they get access? How did that go?
2. Overall, do you think parents should have access to their teenage child’s online medical records?
   1. What are the potential benefits of parents accessing their teenage child’s medical records?
   2. What are the potential downsides?
3. Do you think parents should be able to read their teenage child’s doctors notes?
   1. What are the potential benefits of parents reading these doctors notes?
   2. What are the potential downsides of parents reading these notes?
   3. What is a situation where you would absolutely want your parents to read your doctors notes?
   4. What is a situation where you would definitely **not** want your parents to read your doctors notes?
4. **[If healthy adolescent]** How do you think your views would change if you had a chronic, serious illness?
5. **[If chronically ill adolescent]** How do you think your views would change if you did not have a chronic illness?
6. What do you think your parent thinks? Would they want to have access to your medical records? Your doctors notes?
   1. Do you think your parents have accessed your medical record through the portal?
   2. Do you think they have read any of your doctors notes?
   3. Did you discuss this access with your parents?
      1. [If yes] What was that discussion like?

**[Closing]**

- What advice do you have for other teenagers about accessing their medical records through the portal?

Now, I’d like you to imagine that you are talking to the people at the hospital who decide who gets to access the medical record.

1. What is your advice about providing teenagers with access to their medical records?
2. What is your advice about providing parents with access to their teenage child’s medical records?
3. What is your advice about how to make the online medical record better for families?

Lastly, I’d like you to imagine that you are talking to your doctors.

1. What is your advice about how doctors should talk to families about access to online medical records?
2. What is your advice about how doctors should write their medical notes, knowing that families might read these notes?

Are there any other experiences that you have had, either positive or negative, that you would like to share with us

**Interview Guide Draft – Parent Interviews**

**[Background on 21^st^ Century Cures Act]**

First, I’d like to tell you a little about what we’ll talk about today. Across the country, teenagers can now access their medical records and doctors notes through online portals. At our hospital, we call this portal *MyChart.* These medical records include things like doctors notes, lab results, and imaging results. Parents are given different types of access at different hospitals.

Even though hospitals are offering this access to teenagers, there is no agreed upon best practices. So different hospitals have different rules for how they give access to teenagers and their parents. Today I am going to ask a lot of open-ended questions about your thoughts on this medical record access for teenagers and parents. There are no wrong answers, and you are the expert on your thoughts and beliefs. I just want to learn as much as I can from you.

**[Adolescent’s Health]**

1. To get us started, could you tell me a bit about your child’s health? Does your child have any longstanding medical issues?

***[If so]***

- 1. What types of medical care does your child need?
  2. How many types of doctors does your child see regularly?
  3. How often does your child have medical appointments?
  4. What types of care does your child need at home?

**[EHI Access for Parent]**

1. The online medical record contains lab values, imaging results, appointment reminders, and doctors notes, among other things Have you ever logged into *MyChart* portal and looked at your child’s online medical record?

***[If yes – Reviewed EHI]***

- 1. How did you use the portal? What did you look at?
  2. What made you want you to look at the medical record?
     1. What were you hoping to learn?
     2. Did you learn what you were hoping to learn? How did this information help you?
     3. What problems did this cause for you?
  3. Did your child have to approve your access?
     1. [If yes] Did you talk about this with your child? Could you tell me about that discussion?
     2. [If no] How did you get access to your child’s records?
  4. What made it hard to review the medical record online?
     1. What could have made accessing your child’s online medical record easier?
     2. What could have made the online medical record more helpful for you?
  5. Did you ever read any of the notes from your child’s doctor’s visits?

**[If yes – Yes Notes]**

- - 1. What types of notes did you read?
       1. What made you want to read these notes?
    2. What were you hoping to learn from the notes?
       1. Did you learn what you were hoping to learn? How did this information help you?
       2. What were the downsides of reading these notes?
       3. What could have made these notes more helpful?
    3. Did reading these notes make you look at your team differently?

**[If no – No Notes]**

1. Were you aware that you could read these notes?
2. What kept you from reading the doctors notes?

***[If No – Did Not Review EHI]***

- 1. Were you aware that you could access your child’s online medical record?
     1. What kept you from accessing your child’s online medical record?
     2. Now that you know you can use the portal, do you think you will in the future?
  2. Were there times when it would have been helpful to access your child’s medical record through the portal?
     1. How would this have been helpful?

1. Overall, do you think that parents of teenage patients should be able to access their child’s online medical records?
   1. Do your views change based on the child’s age [**assuming <18 years**]?
   2. What are the potential benefits of parents accessing their teenager’s medical records?
   3. What are the potential downsides of providing parents with this access?
2. Should parents be able to read doctors notes?
   1. What are the potential benefits of parents reading their teenager’s doctors notes?
   2. What are the potential harms of providing parents with this access?
   3. What is a situation where you would absolutely want to read your child’s doctors notes?
   4. What is a situation where you would definitely **not** want to read your child’s doctors notes?
3. **[If healthy adolescent]** How do you think your views would change if your child had a chronic, serious illness like diabetes or sickle cell or inflammatory bowel disease?
4. **[If chronically ill adolescent]** How do you think your views would change if your child did not have [list specific disease]?

**[Access for Adolescent]**

1. Now I want to switch gears and think about teenagers. Has your child accessed their medical records through the portal?
   1. How did they get access? How did it go?
2. Overall, do you think teenage patients should have access to their online medical records? [If so – At what age?]
   1. What are the potential benefits of teenagers having access to their medical records?
   2. What are the potential downsides?
3. Now, what about doctors notes? Do you think teenage patients should be able to read their doctors notes?
   1. What are the potential benefits of teenagers reading their notes?
   2. What are the potential downsides?
   3. What is a situation where you would absolutely want your child to read their doctors notes?
   4. What is a situation where you would definitely **not** want your child to read their doctors notes?
4. **[If healthy adolescent]** How do you think your views would change if your child had a chronic, serious illness?
5. **[If chronically ill adolescent]** How do you think your views would change if your child did not have a chronic illness?
6. Do you think your child has accessed their medical record through the portal?
   1. Do you think they have read any of their doctors notes?
7. Do you think your child wants to have access to their medical records? What about their doctors notes?
   1. Have you ever spoken with your child about this access?
      1. [If yes] What was that discussion like?

**[Closing]**

- What advice would you have for other parents about accessing their teenager’s medical record through the portal?

Now, I’d like you to imagine that you are talking to the people at the hospital who decide who gets to access the medical record.

1. What is your advice about providing teenagers with access to their medical records?
2. What is your advice about providing parents with access to their teenage child’s medical records?
3. What is your advice about how to make the online medical record better for families?

Lastly, I’d like you to imagine that you are talking to your child’s doctors.

1. What is your advice about how doctors talk to families about access to online medical records?
2. What is your advice about how doctors should write their medical notes, knowing that families might read these notes?

Are there any other experiences that you have had, either positive or negative, that you would like to share with us
